# Odds of death after glioblastoma diagnosis in the United States by chemotherapeutic era

**DOI:** 10.1002/cam4.213

**Published:** 2014-03-10

**Authors:** Mitchell S Wachtel, Shengping Yang

**Affiliations:** Department of Pathology and Cancer Center, School of Medicine, Texas Tech University Health Sciences CenterLubbock, Texas

**Keywords:** Bevacizumab, glioblastoma, odds of death, population database, SEER, temozolomide

## Abstract

Bevacizumab (BZM) and temozolomide (TMZ) have been shown to be beneficial in the treatment of patients with glioblastoma. We sought evidence for the benefit of BZM in the general patient population at large. The Surveillance, Epidemiology, and End Results SEER database was queried for patients diagnosed with glioblastoma between 2000 and 2009, divided into a pre-TMZ era (January 2000–June 2003), a transitional era (July 2003–March 2005), a TMZ era (April 2005–October 2007), and a BZM-TMZ era (November 2007–December 2009). Binomial logit regression analyzed odds of death, taking into account age at diagnosis, tumor size, gender, race, marital status, radiotherapy, and extensive surgery. Compared with the pre-TMZ era, odds of death were decreased in the TMZ era by 12% (97.5% CI [confidence interval] 3–20%) 6 months after diagnosis and 36% (30–42%) a year after diagnosis; corresponding values for BZM-TMZ were 31% (24–37%) and 50% (45–55%). For era comparisons, decreases in odds of death were larger at 12 than 6 months; the opposite was true for extensive surgery and radiotherapy (*P* < 0.025, Wald *χ*^2^ test, for each analysis). For both 6 and 12 month comparisons, odds of death in the BZM-TMZ era were lower than in the TMZ era (*P* < 0.025, Wald *χ*^2^ test, for each analysis). The results provide evidence that TMZ positively impacted survival of glioblastoma patients and that the addition of BZM further improved survival, this lends support to the addition of BZM to the chemotherapeutic armamentarium. Evaluation of odds of death is an attractive alternative to Cox regression when proportional hazards assumptions are violated and follow-up is good.

## Introduction

Temozolomide (TMZ) has been shown to benefit patients with glioblastoma when given in association with radiotherapy 1–5. Bevacizumab (BZM) has also shown apparent benefit for treating glioblastoma in academic clinical trials 6–10. Although analyses of population data have shown TMZ to be of benefit 11–16, evaluations of the appropriateness of Cox regression were not made, apart from one which attempted to take proportional hazards (PH) violations into account via a conditional probability model 12; and the effect of BZM was never fully addressed. We hypothesized that the Surveillance, Epidemiology, and End Results (SEER) database 11–15,17 would be able to provide evidence that both drugs have provided benefit to glioblastoma patients in general; moreover, we explored a new approach that, given over 97% follow-up seen in SEER, will likely facilitate analysis of databases for impact of drugs by era.

## Material and Methods

Obtained from SEER via a case listing session from SEER*stat 8.0.4 18 were data from patients with histologically proven glioblastoma (ICD-0-3 9440-9442) diagnosed between 2000 and 2009. All patients were at least 40 years old at diagnosis. No patient had a follow-up of zero. Predictor data were stratified as indicated in Table 1. Era cut points were largely derived from Johnson and O'Neill 13, a variant of which was used by Darefsky, et al. 14: the announcement of the TMZ trial results at the American Society of Clinical Oncology meeting in June 2004 3, FDA approval of TMZ in March 2005 19, and the publication date of the phase II trial result for BZM, October 2007 6. A future analysis might wish to examine results after FDA approval of BZM in November 2009 20. Potential effect modifiers/confounders taken into account, as previously noted, were tumor size, age, extent of surgery, radiotherapy, race, gender, and marital status 12–14.

**Table 1:** Distribution of patients by predictor variable

Pre-temozolomide era (January 2000–June 2003)	6631 (31.8%)
Transitional era (July 2003–March 2005)	3692 (17.7%)
Temozolomide era (April 2005–October 2007)	5562 (26.6%)
Bevacizumab-temozolomide era (November 2007–December 2009)	4994 (23.9%)
	20,879 (100.0%)
70+ years old	6641 (31.8%)
55–69 years old	8405 (40.3%)
40–54 years old	4690 (22.5%)
18–39 years old	1143 (5.5%)
	20,879 (100.0%)
First primary	2709 (13.0%)
Not first primary	18,170 (87.0%)
	20,879 (100.0%)
5+ cm tumor	6690 (32.0%)
<5 cm tumor	9324 (44.7%)
Unknown tumor size	4865 (23.3%)
	20,879 (100%)
Widowed	2187 (10.5%)
Divorced or separated	1875 (9.0%)
Single	2478 (11.9%)
Married, including common law	13,764 (65.9%)
Unknown	575 (2.8%)
	20,879 (100.0%)
Male	12,274 (58.8%)
Female	8605 (41.2%)
	20,879 (100.0%)
White	18,937 (90.7%)
Black	1100 (5.3%)
Other (Asian/Pacific Islander/Native American)	842 (4.0%)
	20,879 (100.0%)
Radical resection or lobectomy (extensive surgery)	6527 (31.3%)
Less than extensive surgery	14,079 (67.4%)
Unknown	273 (1.3%)
	20,879 (100%)
Radiotherapy administered	4273 (20.5%)
Not done	16,146 (77.3%)
Unknown	460 (2.2%)
	20,879 (100.0%)

Analyses employed R 3.0.1 21, with its boot 22 and survival 23 routines. The Fleming–Harrington method generated 1-year survivals. Cox regression estimated hazard ratios (HR). Because era, age, tumor size, surgery, and radiotherapy all showed PHs assumption violations with *P* < 0.0001 by Therneau–Grambsch tests, binary logit regressions were applied to calculate odds ratios (OR) at 6 and 12 months after diagnosis. A Bonferroni adjustment was made to adjust for multiple testing, thus null hypotheses were rejected when *P* < 0.025. The 1.25th and 98.75th percentiles of 10,000 bootstrap replicates were deemed 97.5% confidence intervals (97.5% CI); bootstrap standard errors were also calculated for use in Wald *χ*^2^ tests to compare 6 and 12 month ORs and evaluate differences between eras.

## Results

Table 1 provided distributions of patients by predictor variable. A year after diagnosis 13,259 of the 20,879 (63.5%) patients were deceased, with 1-year survival estimates of 31.8% (97.5% CI 30.6–33.0%) for the Pre-temozolomide (pre-TMZ) era, 37.3% (35.6–39.0%) for the transitional (TR) era, 41.0% (39.6–42.5%) for the temozolomide (TMZ) era, and 43.0% (41.4–44.4%) for the bevacizumab-temozolomide (BZM-TMZ) era, demonstrating progressive improvement in survival by drug era. Although the greatest difference lay between the first two eras, such general statements have often proven unwise without taking into account modifying/confounding variables; advances in age over time, for example, might have rendered the latter two eras less prognostically different than they appear to be. The taking into account of effect modifiers/confounders has always been an important purpose of multivariate regression.

The most commonly used multivariate regression analysis for survival has been Cox regression, which estimates HR of the hazard rates corresponding to the conditions described by two levels of an explanatory variable. HRs for all variables of interest, as estimated by multivariate Cox regression, were placed in the left column of Table 2. An example of how to interpret an HR: for a 20-year-old and a 90-year-old patient, assuming all other things being equal, the 0.22 HR implied the former faced about one-fifth the hazard of death of the latter. Note the diagnostic eras were, on multivariate regression, more evenly distributed than the univariate assessment indicated; the HR for each succeeding era changed by a factor of 0.90. Patients who underwent surgery or received radiotherapy appeared, by evaluation of the HR, to have an even more dramatic change in fate than was apparent on univariate analysis. Having a small tumor, not being widowed, being a woman, and being Asian also appeared to positively impact survival. Because the 97.5% CI's for the associated HR's do not include 1, the differences were deemed to not have been explicable by chance (*P* < 0.025).

**Table 2:** Results of Cox regression and tests of proportional hazards assumption; not displayed are comparisons with unknown

Comparison	Results of regression HR (97.5% CI)	Therneau–Grambsch tests *ρ* (*χ*², *P*)
TR vs. Pre-TMZ era	0.89 (0.85–0.94)	−0.017 (5.7, 0.0169)
TMZ vs. Pre-TMZ era	0.82 (0.78–0.86)	−0.032 (19.2, <0.0001)
BZM-TMZ vs. Pre-TMZ era	0.72 (0.69–0.76)	−0.021 (8.21, 0.0042)
55–69 years vs. 70+ years	0.57 (0.55–0.06)	0.032 (19.0, <0.0001)
40–54 years vs. 70+ years	0.39 (0.37–0.42)	0.081 (120, <0.0001)
18–39 years vs. 70+ years	0.22 (0.20–0.24)	0.055 (57.8, <0.0001)
No prior cancer vs. prior	0.96 (0.91–1.01)	−0.009 (1.68, 0.195)
<5 vs. 5+ cm tumor	0.89 (0.85–0.92)	0.036 (24.9, <0.0001)
Divorced vs. widowed	0.92 (0.84–1.01)	−0.004 (0.25, 0.615)
Single vs. widowed	0.87 (0.80–0.95)	−0.001 (0.03, 0.853)
Married vs. widowed	0.80 (0.75–0.85)	0.010 (1.90, 0.168)
Women vs. men	0.94 (0.91–0.98)	−0.020 (7.69, 0.0055)
Black vs. white	1.02 (0.94–1.11)	−0.007 (0.85, 0.356)
Other vs. white	0.86 (0.78–0.94)	0.002 (0.12, 0.735)
Extensive surgery vs. not	0.68 (0.66–0.71)	0.105 (207, <0.0001)
Radiotherapy vs. not	0.49 (0.46–0.52)	0.233 (969, <0.0001)

CI, confidence interval; HR, hazard ratios; TR, transitional; TMZ, temozolomide; BZM, bevacizumab.

For the posited 20- and 90-year-old patient pair, an HR of 0.22 implied the latter was about five times more likely than the former to die if both have survived 2 months, 10 months, or even 3 years after a diagnosis of glioblastoma; this assumption that the HR is independent of time since diagnosis has been called the PHs assumption. Residuals have been said, in general, to express differences between observed and expected results; for Cox regression the vital residuals have always been scaled Schoenfeld residuals. To test PH, Therneau–Grambsch tests generated rho (*ρ*) and chi-square (*χ*^2^) values to discern a correlation between scaled Schoenfeld residuals and time. Results of PH tests in the right column of Table 2 rendered questionable all but the marital and racial assertions of the prior paragraph; because all other statistically significant HR bore correlations between residuals and time that were deemed to not have been explicable by chance (*P* < 0.025 for each assessment).

**Figure 1:**
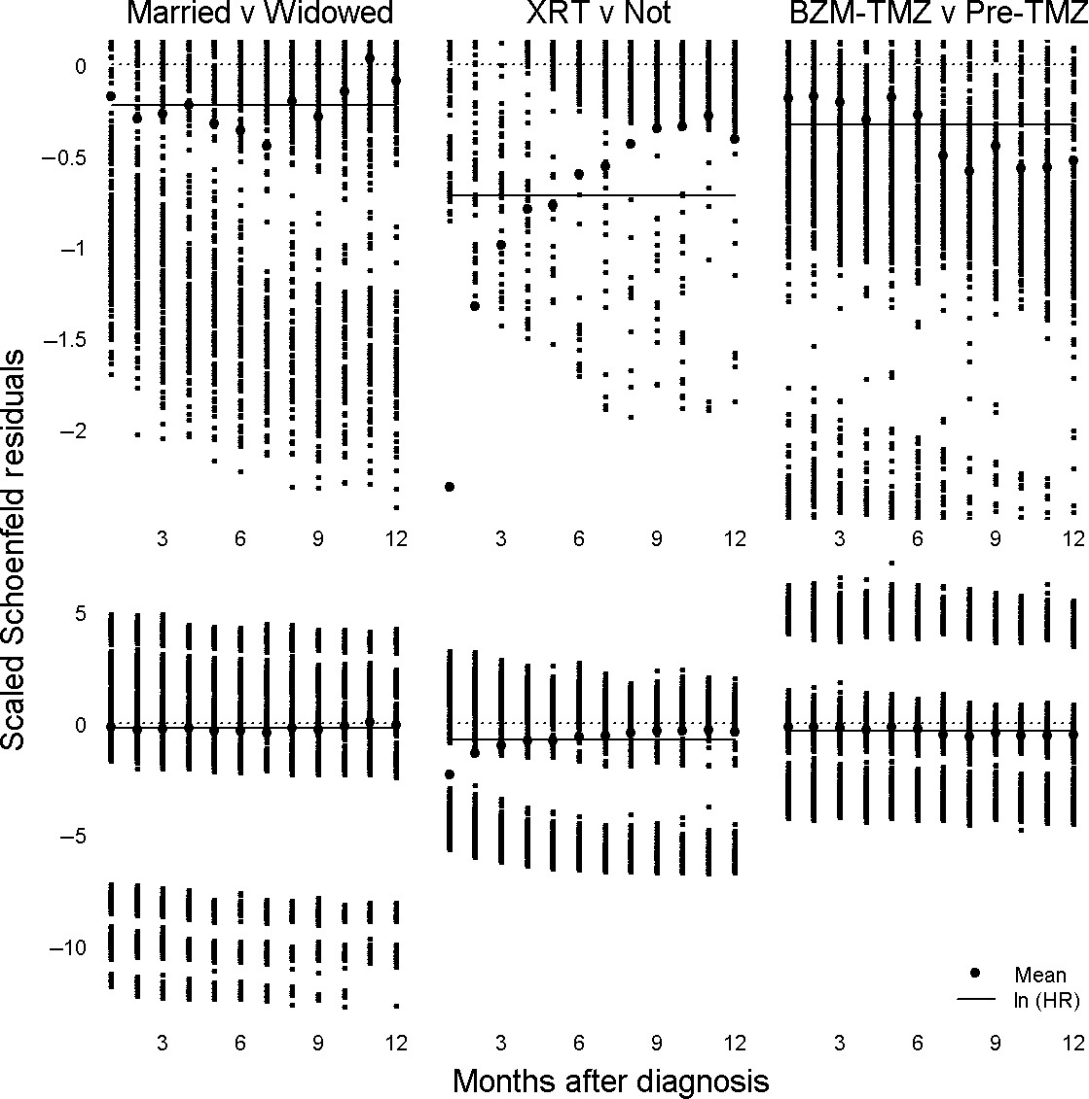
Scatterplots of residuals and time for Cox regression, top plots being regions of interest of bottom plots.

Plotting scaled Schoenfeld residuals against time, as demonstrated for the three comparisons in the three columns of Figure 1, has proven a vital assistant in assessing the importance of PH assumption violations. For each comparison, the upper graph was a magnified view of the region of interest in the lower graph, which included all scaled Schoenfeld residuals for the first year. For each month, thousands of deaths were recorded, such that the individual results were joined together mostly in apparent segments. The discrete nature of the residuals as respects time, generated by SEER's recording survival in months and not days, showed a minor reason to avoid Cox regression, which assumes data are not broadly grouped. At any point in time, the mean of the scaled Schoenfeld residuals was the natural logarithm of the HR; solid horizontal lines in each graph lay at the natural logarithms of calculated HR values, ln(0.80) for married versus widowed, ln(0.49) for radiotherapy administered versus radiotherapy not administered, and ln(0.72) for BZM-TMZ era versus pre-TMZ era. Each large black dot was a mean of a month's Schoenfeld residuals. Perusal of the bottom graphs shows that, apart from month 1 for the radiotherapy comparison, the natural logarithms of the estimated HR's were what one might expect, being about midway between the highest and lowest large dots. PH problems were made apparent by the upper graphs.

For the marital status comparison, the left graph pair of Figure 1, PH was not shown to be violated; the large dots lay above and below the solid line in a random fashion consistent with the notion that the relative advantage married people possessed over the widowed did not change with time. The radiotherapy comparison, the middle graph pair of Figure 1, showed a large advantage to radiotherapy in the first month that rapidly declined, crossing the HR line by month 6, consistent with radiotherapy's largely having been administered soon after diagnosis. By contrast, the era comparison, the right graph pair of Figure 1, gave a much greater advantage to BZM-TMZ in the second half of the year, crossing the HR line by month 7, consistent with BZM's being used for salvage therapy because treatment failure implies a passage of time. For radiotherapy and era comparisons, observed changes in HR thus likely reflected the nature of the therapy being provided; the problem is that then the calculated HR were precluded from general discourse because the hazard at 1 month was not the same as it is as 12 months or, for that matter, any other month. Because era differences were the focus of the study, Cox regression was contraindicated, as were almost all other survival analyses, which except for lognormal accelerated failure time regression, required PH not be violated. Although lognormal accelerated failure time adjusted all variables for a change as respects time, it required a stronger assumption on survival time. Time-dependent covariates were precluded due to complexity; other graphs showed nonlinear changes in HR. Moreover, analyses with time-dependent covariates would have only been able to say what the instantaneous risk was at a particular point in time; an estimated 6 month HR in a model with time-dependent covariates would not in itself say anything to a clinician with a patient who has just been diagnosed about the likelihood of death within 6 months.

**Figure 2:**
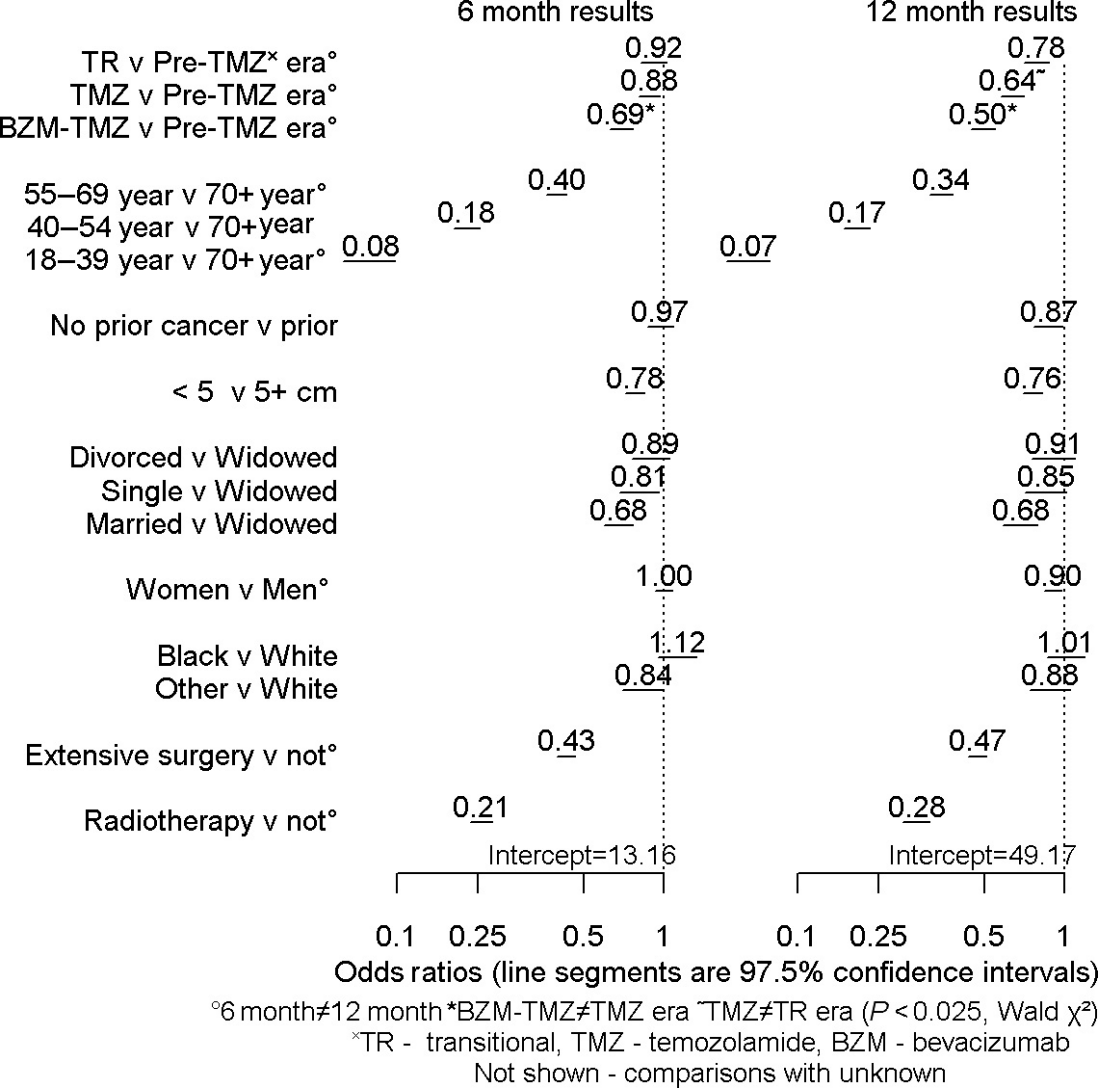
Results of binomial logit regressions.

To accurately assess era differences, a reasonable approach lay in binary logit regression, which calculated OR; this was so in part because only 174 (0.8%) of the 20,879 patients were lost to follow-up at 1 year. Two times after diagnosis, 6 and 12 months, were chosen for analysis because median survival for all eras was under a year. Figure 2 displayed results of binary logistic regression analyses. Compared with the pre-temozolomide (pre-TMZ) era, adjusted odds of death were decreased in the TMZ era by 12% (97.5% CI 3–20%) 6 months after diagnosis and 36% (30–42%) a year after diagnosis; corresponding values for the BZM-TMZ era were 31% (24–37%) and 50% (45–55%). For all era comparisons, decreases in odds of death were larger at 12 than at 6 months; the opposite was true for surgery and radiotherapy. For both 6- and 12-month comparisons, odds of death in the BZM-TMZ era were 22% lower than in the TMZ era. Age showed the largest impact; each added decade of life had an associated large increase in adjusted odds of death; there was a difference seen between 6 and 12 month results for 55–69 years and 18–39 years, but not for 40–54 years. The odds of death for patients with tumors smaller than 5 cm were three-fourths those of patients with larger tumors. Patients without a prior tumor diagnosis did not display different ORs at 6 months, but by 1 year they showed 13% lower odds of death than patients with a prior cancer diagnosis. Being widowed was similar to being divorced or separated; being single was associated with lower odds of death at six, but not 12 months. Married persons had two-thirds the odds of death of widowers at both time periods. Women had a small advantage over men at 1 year, but not 6 months. Although blacks and whites did not differ, Asians/Pacific Islanders/Native Americans had lower odds of death than Whites at 6, but not 12 months.

All groups on the right of the “v”'s in Figure 2 were provided 1 for their coefficient. Armed with this knowledge, estimation of odds of death for any particular group of interest lay simply in multiplying appropriate numbers in the figure. The 6-month odds of death for white married women, 40–54 years, in the TZM era who had undergone extensive surgery and radiotherapy, had small tumors and lacked prior cancer diagnoses, odds of death were:


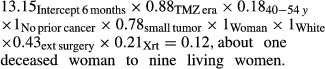


The probability of death was calculated by dividing the odds by (odds + 1) and multiplying by 100. Thus, the probability that a patient in this group would be dead within 6 months was (0.12/1.12) × 100 = 10.7%.

Assumptions of generalized linear models were not shown to be violated for binomial logit regression models. *T-*tests of standardized residuals did not show that, on average, they differed from 0 (*P* > 0.05). F tests of regression of fitted values upon standardized residuals (*P* > 0.05) and squared standardized residuals (*P* > 0.05) did not show dependence of residuals upon expected values or heteroskedasticity. There were no influential outlier groups.

## Discussion

Given excellent follow-up for cancer registries, binomial logit regression was quite advantageous. Although the analysis was restricted to the follow-up of the last set of patients studied, limiting matters to a 12-month postdiagnosis analyses, this was more than compensated by the lack of a requirement for time-invariant HRs. Assumptions of this analysis were merely those of almost any other regression analysis. Results were then reported in terms quite meaningful as respects relative odds of death at one or more specific times of interest. Because median survival for all eras was under a year, 6-and 12-month assessments proved quite valuable. The study provided reason to think TMZ and BZM had positive effects on survival that increased with time after diagnosis, in contrast to the apparently diminishing response to radiotherapy and surgery. Another advantage to the use of this method is that it avoids a problem called immortal time bias, which is a failure to take into account, in calculation of general HRs, the time of adoption of radiotherapy 24. Needless to say, this caveat applies with even greater force when one is dealing with chemotherapy that is administered to some patients for a much longer period than others. The bootstrap deserves special emphasis, as it permits the investigator to simultaneously compare paired results from two separate analyses while calculating CIs of ORs. Because an intercept is provided, the need to provide tables of survivals is eliminated, in that any particular interest a reader might have can be satisfied by simple multiplication.

In the setting of an academic trial, TMZ 1–5 and BZM 6–10 proved an excellent chemotherapeutic agent in the context of radiotherapy for glioblastoma. A meta-analysis even suggested the drug may be of use without radiotherapy in the setting of the elderly population 25. Although population-based analyses of had shown an advantage to TMZ 11–16, advances in survival have not yet been demonstrated by BZM; Cox regression was used with very little question being asked of its appropriateness, which, as shown here, is indeed an issue. One possible result was evidenced by the publication of tables of median survivals by age group 14, a univariate examination that cannot take into account the other variables affecting survival. Although conditional probability avoids PHs matters to some degree 12, the changes in HRs in the present case were large enough that that would not completely resolve the matter. This study avoided repetitive analysis in Johnson and O'Neill 13 by using a single variable set to compare odds of death at each time period of interest with one another. The bootstrap enabled direct calculations of CIs of changes from one era to another, which regression alone could not accomplish.

We compared the results of our analyses with several major studies on the topic. The 0.64 OR advantage conferred by TMZ era is nearly identical to the 0.6 HR advantage accorded TMZ in the 5-year retrospective study of Stupp of EORTC 2. The 0.64 HR benefit accorded BZM in a 2012 study 26 has a 95% ci that includes the 0.78 OR seen here; the difference also reflects the data analyzed in this study being from a TR era, largely before FDA approval. Moreover, there exists a limit with respect to analysis of BZM, in that a wide array of therapies have recently been proposed 6,27,28 that make era analysis less appropriate for assessment of the efficacy of BZM. This study showed that age group bore an increased risk, something that should be taken into account when therapy is adjusted for age with respect to glioblastoma; the results reflect the findings of the Radiation Oncology Therapy Group 29, but expand their findings by showing that persons 70+ years are at greater risk of death than any other group. As with other studies, tumor size made a difference, although the magnitude was less than that of Daniels, et al. 30 who found, in comparing tumors larger than 6 cm a 1.85 HR. The general conclusion would be that when age is considered for the making of therapeutic decisions, the risk be assessed for patients in general, not simply those above a certain cut point. It must be remembered that this assessment uses a surrogate for chemotherapeutic protocols, the time periods of approval of these therapies; given the importance of changes in drug therapy to researchers everywhere, consideration in the SEER database should be given to provisioning data on specific drug regimens.

In summary, this study provided evidence that TMZ positively impacted survival and that adding BZM to the regimen further improved survival among US glioblastoma patients in general. Because near-complete follow-up characterizes modern databases, binary logit regression is an attractive alternative to Cox regression when PHs violations are deemed important.
